# Designing a preliminary adaptive study to inform a biomarker trial in Psoriasis

**DOI:** 10.1186/1745-6215-12-S1-A17

**Published:** 2011-12-13

**Authors:** Andrew T Prevost, Jack Bowden

**Affiliations:** 1 Department of Primary Care and Public Health Sciences, King’s College London, SE1 3QD, UK; 2 Medical Research Council Biostatistics Unit, Institute of Public Health, Cambridge, CB2 0SR, UK

## Background

Biomarkers play different roles in trials, being accordingly classified into ‘prognostic’, ‘predictive’, ‘surrogate’, or combinations thereof. Knowledge of a biomarker’s role enables focused testing in late Phase trials through a better choice from available designs (e.g. ‘stratified’, ‘strategy’, and ‘enrichment’). Preliminary studies can inform a biomarker’s role and the relative value of multiple biomarkers.

## Motivation

Here we consider the design of a preliminary study of potentially predictive biomarkers in patients treated for Psoriasis. A clinical researcher came for methodological advice, bringing a related published study with exciting results but of highly dubious quality. The objective was to provide a design with better properties (less bias, high power, low cost), allowing multiple biomarkers and their combination to be assessed to inform any subsequent trial.

## Methods

Prior preferences, agreed by the researcher, were for a prospective design, control groups, and power-based sample size calculation. A formal 10-minute presentation to the full team was required to explain pros and cons of an adaptive element (two recruitment stages) to the design. Power was assessed through simulation in R-software using Fisher’s method, involving the product of stage p-values.

## Results

Effect size was defined in terms of the correlation between treatment response over time and a biomarker. Under a non-adaptive design, an R-squared of 20% could be detected with 90% power, 5% significance level, with 49 patients, with all 17 expensive biomarkers measured. The adaptive design offered an interesting alternative, employing p>0.3 to discontinue with biomarkers quarter-way through recruitment, requiring 24+72=96 patients. This offers more patients to develop a combination from an enriched biomarker set guaranteed to include the best five performers from stage one. The proportion of biomarkers expected to discontinue, conditional on underlying effect size, was considered graphically.

## Conclusions

Incorporating methodological improvements into study designs requires understanding of methodology and collaborators. The cost-efficient two-stage design is an improvement on the related published study, and we outline further analysis-stage developments: reducing bias in estimates and providing valid confidence intervals and error rates [1,2]. The study proposal is currently going through ethics.
